# TEM and STEM Studies on the Cross-sectional Morphologies of Dual-/Tri-layer Broadband SiO_2_ Antireflective Films

**DOI:** 10.1186/s11671-018-2442-4

**Published:** 2018-02-12

**Authors:** Shuangyue Wang, Hongwei Yan, Dengji Li, Liang Qiao, Shaobo Han, Xiaodong Yuan, Wei Liu, Xia Xiang, Xiaotao Zu

**Affiliations:** 1 Institute of Fundamental and Frontier Sciences, University of Electronic Science and Technology of China, Chengdu, 610054 People’s Republic of China; 2 School of Physical Electronics, University of Electronic Science and Technology of China, Chengdu, 610054 People’s Republic of China; 3 Dalian National Lab for Clean Energy Dalian Institute of Chemical Physics, Chinese Academy of Science, Dalian, 116023 China; 4 China Academy of Engineering Physics, Mianyang, 621900 China

**Keywords:** Sol–gel process, Antireflective film, TEM, STEM, Density ratio

## Abstract

Dual-layer and tri-layer broadband antireflective (AR) films with excellent transmittance were successfully fabricated using base-/acid-catalyzed mixed sols and propylene oxide (PO) modified silica sols. The sols and films were characterized by scanning electron microscope (SEM), Fourier transform infrared spectroscopy (FTIR), nuclear magnetic resonance (NMR), transmission electron microscope (TEM), and scanning transmission electron microscope (STEM). FTIR and TEM results suggest that the PO molecules were covalently bonded to the silica particles and the bridge structure existing in PO modified silica sol is responsible for the low density of the top layer. The density ratio between different layers was measured by cross-sectional STEM, and the results are 1.69:1 and 2.1:1.7:1 from bottom-layer to top-layer for dual-layer and tri-layer films, respectively. The dual-layer film demonstrates good stability with 99.8% at the central wavelength of 351 nm and nearly 99.5% at the central wavelength of 1053 nm in laser system, and for the tri-layer AR film, the maximum transmittance reached nearly 100% at both the central wavelengths of 527 and 1053 nm.

## Background

Broadband antireflective (AR) films have been widely used in optical devices such as automotive windows, solar cells, laser systems, and many energy-related applications to increase the availability of light [1–7]. To prepare a good AR film, it is necessary to control film thickness and its optical reflective index, which must satisfy the following principle: thickness of the film should be λ/4, where λ is the wavelength of the incident light, and

1 $$ {\boldsymbol{n}}_{\boldsymbol{c}}={\left({\boldsymbol{n}}_{\boldsymbol{a}}\times {\boldsymbol{n}}_{\boldsymbol{s}}\right)}^{\mathbf{0.5}} $$

where *n*_c_, *n*_a_, and *n*_s_ are the refractive indices of the film, air, and substrate, respectively [8, 9].

Generally, broadband AR films can be fabricated by traditional methods, such as lithography [10, 11], layer-by-layer assembly [12, 13], block copolymer phase separation [14, 15], and sol–gel methods [16–18]. Among them, the sol–gel method has attracted much interest due to its low cost, simple operation process, controllable microstructure, and easy large-volume production on a substrate, regardless of the shape or size of the surface. However, the drawback is that it is specific to only one wavelength and accompanied by a V-shape reflection spectrum, resulting in a dramatic decrease of transmittance upon deviating from the peak position. In many situations, especially in laser system, the process of conversion of 1053 nm laser to 351 nm laser imply that, in some case, there exist simultaneously laser beams at 351, 527, and 1053 nm passing through an optical component, and an AR film effective simultaneously at two or three wavelengths is highly desirable. Dual-layer or tri-layer broadband antireflective (AR) film is a good solution to meet the laser system demands. According to Eq. 1, the key parameter for preparing dual- and tri-layer films is to adjust the refractive index of each layer. The porosity (or in other words, the density) of each layer has significant influence on the refractive index as demonstrated by previous research [18–20]. According to the effective medium theory, the refractive index of porous materials is given by [21].

2 $$ {\boldsymbol{n}}_{\boldsymbol{p}}^{\mathbf{2}}=\left({\boldsymbol{n}}_{\boldsymbol{s}}^{\mathbf{2}}-\mathbf{1}\right)\left(\mathbf{1}-\boldsymbol{p}\right)+\mathbf{1} $$

where *n*_p_ and *n*_s_ refer to the refractive index of the porous material and solid material, respectively, and *p* is the porosity of a porous material. However, it is difficult to measure the pore size, grain size, and porosity of the film as the thickness is only tens to a hundred of nanometers. Most of the reported porosity measurement methods are calculated or analogical. For example, Orignac et al. [22] reported the porosity volume fraction *V*p is estimated as the ratio between the sum of the areas of the pores measured in the SEM image and the total imaging area of the sample. Xiao et al. [23] measured the reflective index based on the relationship between the reflective index and acid- or base-catalyzed sol ratio. They found the refractive index of the mixed AR films is proportional to the acid- to base-catalyzed sol ratio. With an acidic catalyst, the growth of silica sol tends to form linear chains, giving the acid-catalyzed AR film a refractive index of 1.44. By mixing the base-catalyzed and acid-catalyzed silica sols together, AR film with refractive index varying from 1.22 to 1.44 can be prepared. Ye et al. [24, 25] reported another method to measure the porosity of the films based on Brunauer–Emmett–Teller’s **(**BET) surface area test method. In order to quantitatively demonstrate the porosity of the films, the xerogel powders were produced under a similar condition to the fabrication of films, so The BET data of these xerogel powders should be close to the actual properties of the corresponding films to some extent. Although this method can be used to approximately calculate the porosity of the film, it is difficult to verify the data error between the film and the xerogel powders.

In this work, the cross-sectional morphologies of the dual-/tri-layer films were characterized by SEM and TEM. The sizes of pores and silica grains of each two layers were analyzed. The results show that the sizes of pores as well as the silica grains were increased from the bottom to the top layer. In addition, there is an apparent interface between two layers. The density ratio from the bottom to top film in dual-/tri-layer film was measured by a dark-filed STEM, according to the element signal intensity. The density ratio is 1.69:1 and 2.1:1.7:1 for dual-layer and tri-layer films, respectively. Firstly, the dual-layer and tri-layer broadband AR films were prepared by a sol–gel process via pulling method. The bottom layer was prepared by mixing the acid-catalyzed and base-catalyzed silica sols, and the top layer was prepared from PO modified silica sol according to literature reports [26]. The sols were characterized by TEM, FTIR spectrum, and NMR spectrum. The results revealed that the PO molecules were covalently bonded to the silica particles and the bridge structure existing in PO modified silica sol contributed to the low density of the top layer. The dual-layer silica film showed a simultaneously high transmittance at wavelengths of 351 nm laser and 1053 nm laser. Moreover, the film showed good stability. After 63 days, there was no obvious difference compared with the initial spectrum.

## Methods/Experimental

### 2.1 Preparation of Silica Sol

The process of the preparation of different sols are based on the literature reports [26], following below:

#### 2.1.1 Preparation of Base-Catalyzed Silica Sol (Sol A)

Tetraethyl silicate (164 g) was mixed with anhydrous ethanol (1385 g), ammonia water (25–28%) 8.7 g, and deionized water (10 g). The solution was set in a closed glass container and stirred at 30 °C for 2 h and then aged at 25 °C for 7 days. It was then refluxed for more than 24 h to remove ammonia. This yielded a 3% by weight base-catalyzed sol of silica in ethanol, and this was finally filtered through a 0.22-lm PVDF membrane filter prior to use.

#### 2.1.2 Preparation of Acid-Catalyzed Silica Sol (Sol B)

Tetraethyl silicate (104 g) was mixed with anhydrous ethanol (860 g) and water (36 g) that contained concentrated hydrochloric acid (0.2 g). The solution was left in a closed glass container and stirred at 30 °C for 2 h and then aged at 25 °C for 7 days. This yielded a sol of acid-catalyzed silica in ethanol with an equivalent silica concentration of 3%. It was also filtered through a 0.22-μm PVDF membrane filter prior to use.

#### 2.1.3 Preparation of Base-/Acid-Catalyzed Mixed Sol (Sol C)

The 3% based-catalyzed silica sol and the 3% acid-catalyzed silica sol were mixed in proportions to prepare acid-catalyzed silica in total silica of 0–80% and stirred at 30 °C for 2 h.

#### 2.1.4 Preparation of PO Modified Silica Sols (Sol D)

Tetraethyl silicate (164 g) was mixed with anhydrous ethanol (1385 g), ammonia water (25–28%) 8.7 g, and deionized water (10 g), and then, 0.92, 1.84, 2.76, 3.64, 4.6, 7.36, and 9.2 g PO were also added into the mixed solution to give PO weight ratio to silica of 2–20%, respectively. The final solution was left in a closed glass container and stirred at 30 °C for 2 h and then aged at 25 °C for 14 days.

### 2.2 Preparation of AR Film

The fused silica substrates were ultra-sonicated in acetone for 10 min and wiped carefully using clean room wipers. For dual-layer silica AR film, Sol C and Sol D were deposited on well-cleaned fused silica substrates by dip film, respectively. The thickness of each film was finely tailored by tuning the withdraw rates. The films were heat-treated at 160 °C for 8 h under ambient atmosphere. The tri-layer silica AR films were prepared according to the reports by Ye et al. [24] briefly. The PVDF-modified base-catalyzed silica sol was used for the middle layer of the three-layer film. The mixture of PVDF-modified base- and acid-catalyzed was used for the bottom layer. The final ORMOSIL sol was named as Sol E, which was used for the top layer of the three-layer film.

Microstructures and morphologies of silica sols and AR films were characterized by microstructures and morphologies of silica sols, and AR films were characterized by Fourier transform infrared spectroscopy (FTIR, IRTracer100), nuclear magnetic resonance (NMR, EchoMRI-500), scanning electron microscope (SEM, JEOL JSM-7001F at 15 kV), and transmission electron microscope (TEM, JEM-2010FEF). Selected area electron diffraction (SAED) was also recorded using the same equipment.

## Results and Discussion

### 3.1 Characterizations of Silica Sols

**Fig. 1:**
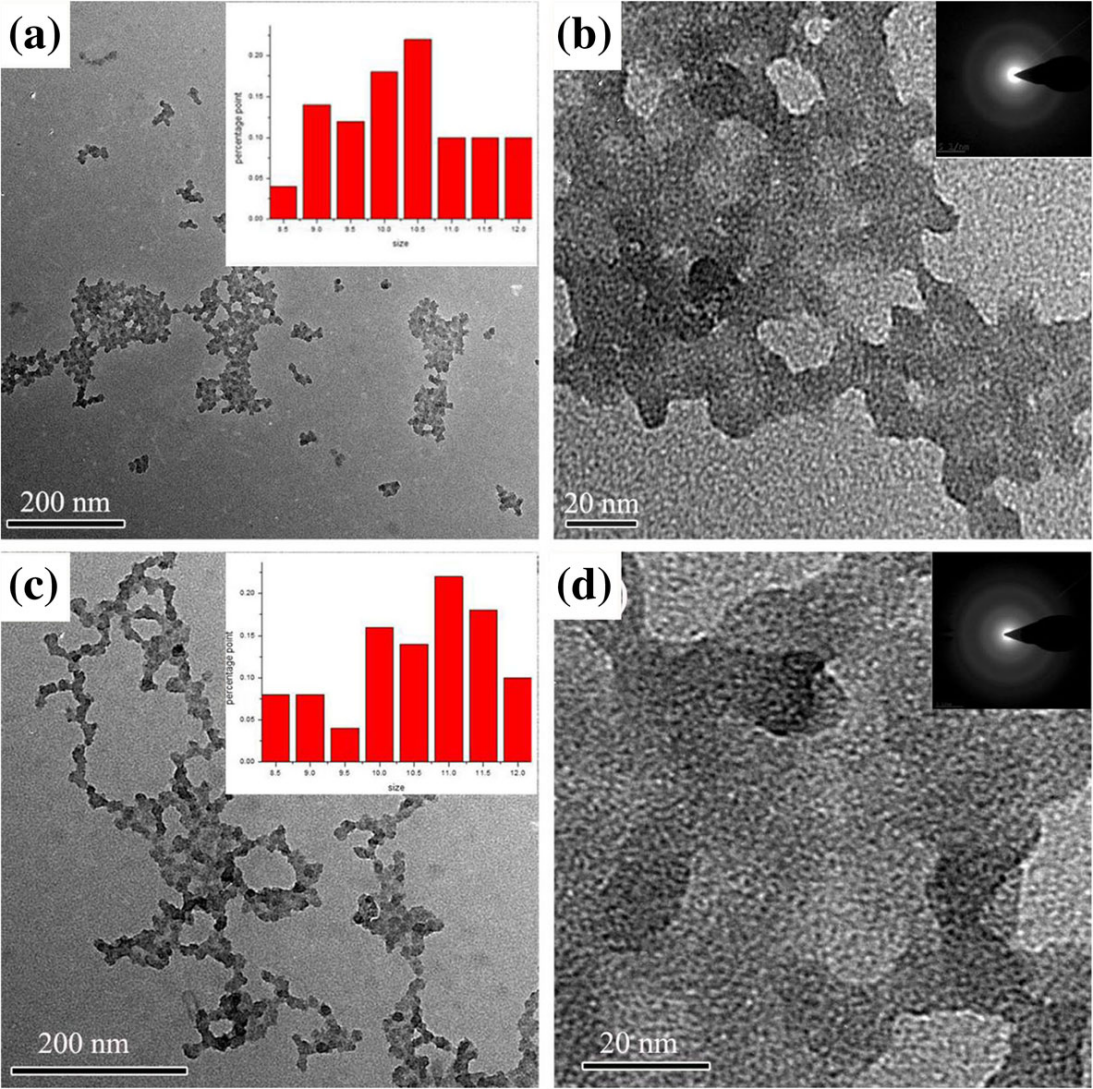
**a** Low-magnification TEM images of silica sol C. **b** High-magnification TEM images of silica sol C. **c** Low-magnification TEM images of silica sol D. **d** High-magnification TEM images of silica sol D. Insets in images are the corresponding grain size distribution histogram and SEAD spectrum

Particle size and its distribution are important properties for silica sols as they determine the final characters of the AR films. The TEM images of the silica sol are shown in Fig. 1a, b, respectively. Obvious aggregation can be seen between sol particles. The inserted size distribution histogram shows an average size of 10 nm. SAED spectrum (insert of Fig. 1b) indicates the particles are amorphous. Figure 1c, d shows the TEM images of PO modified silica sol. The silica particles were aggregated and the borders were fogged, suggesting the existence of some connections among the silica particles [27, 28]. The result is in agreement with the previous reports [27, 29]. As PO contains hydroxyl groups, a similar “bridge structure” is responsible for the bigger particle formation in the sol. When PO is added, there are some chemical links formed between SiO_2_ particles. The “PO bridges” can be linked by C–O–Si bonds or hydrogen bonds [26]. The range of particle size (inserted in Fig. 1d) is between 9 and 13 nm, larger than that of the particles without PO. A larger particle leads to a lower film density and hence a lower refractive index [29].

**Fig. 2:**
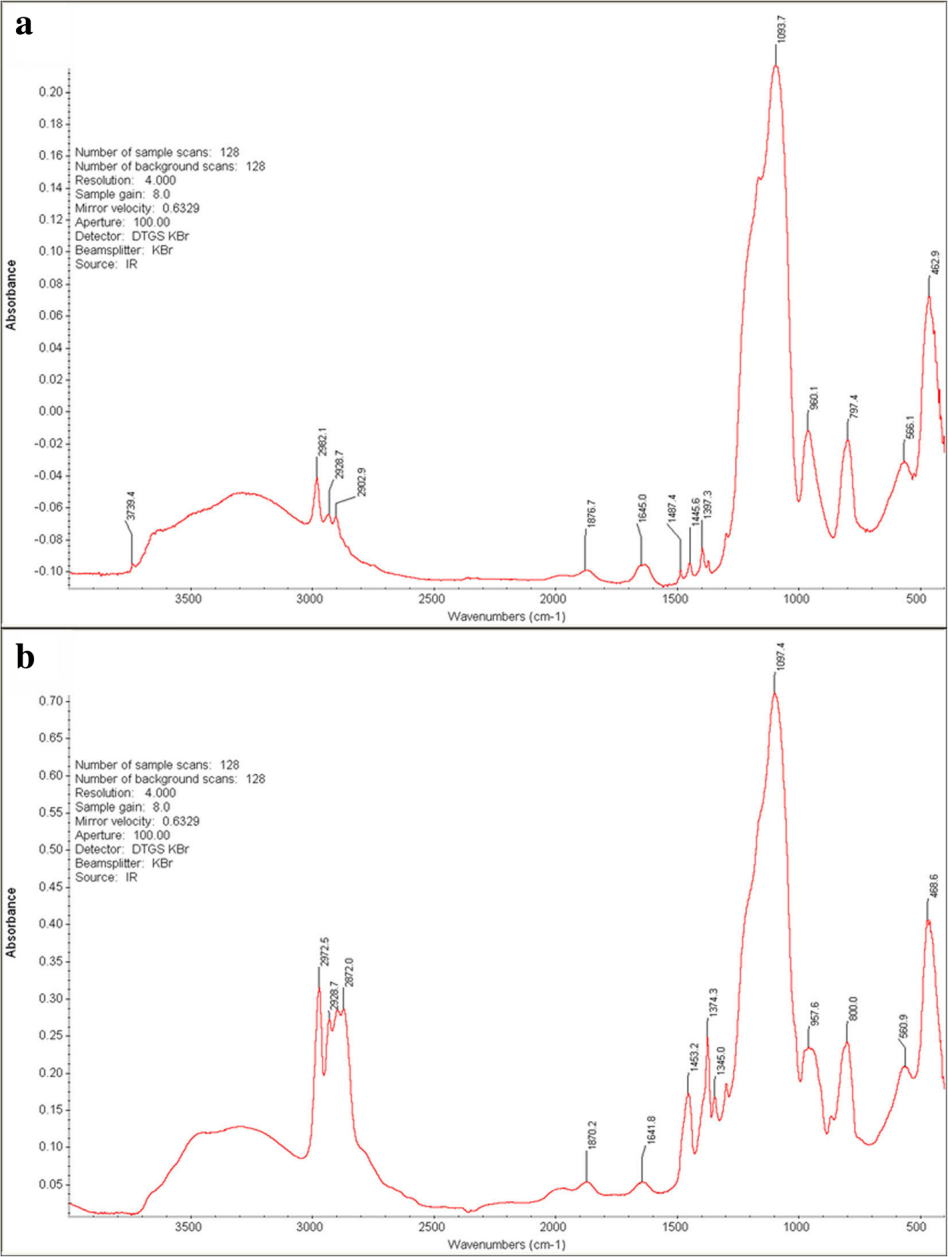
**a** FTIR spectrum of silica sol C. **b** FTIR spectrum of silica sol D

The FTIR spectra of the obtained sol C and sol D are shown in Fig. 2. The absorption peaks at 1099 and 800 cm^−1^ (appearing in both spectrums) were assigned to Si–O–Si anti-symmetric and symmetric stretching vibrations, indicating the existence of the silica particles. The absorption peak at 962 cm^−1^ was assigned to stretching vibration of Si–OH. In addition, the absorption peak at 1278 cm^−1^ was assigned to stretching of the C–O bond, and the absorption peaks at 2972, 2928, and 2872 cm^−1^ (Fig. 2b) were assigned to the vibration of alkyl groups in the PO molecules, suggesting that the PO molecules were covalently bonded to the silica particles [23, 30].

**Fig. 3:**
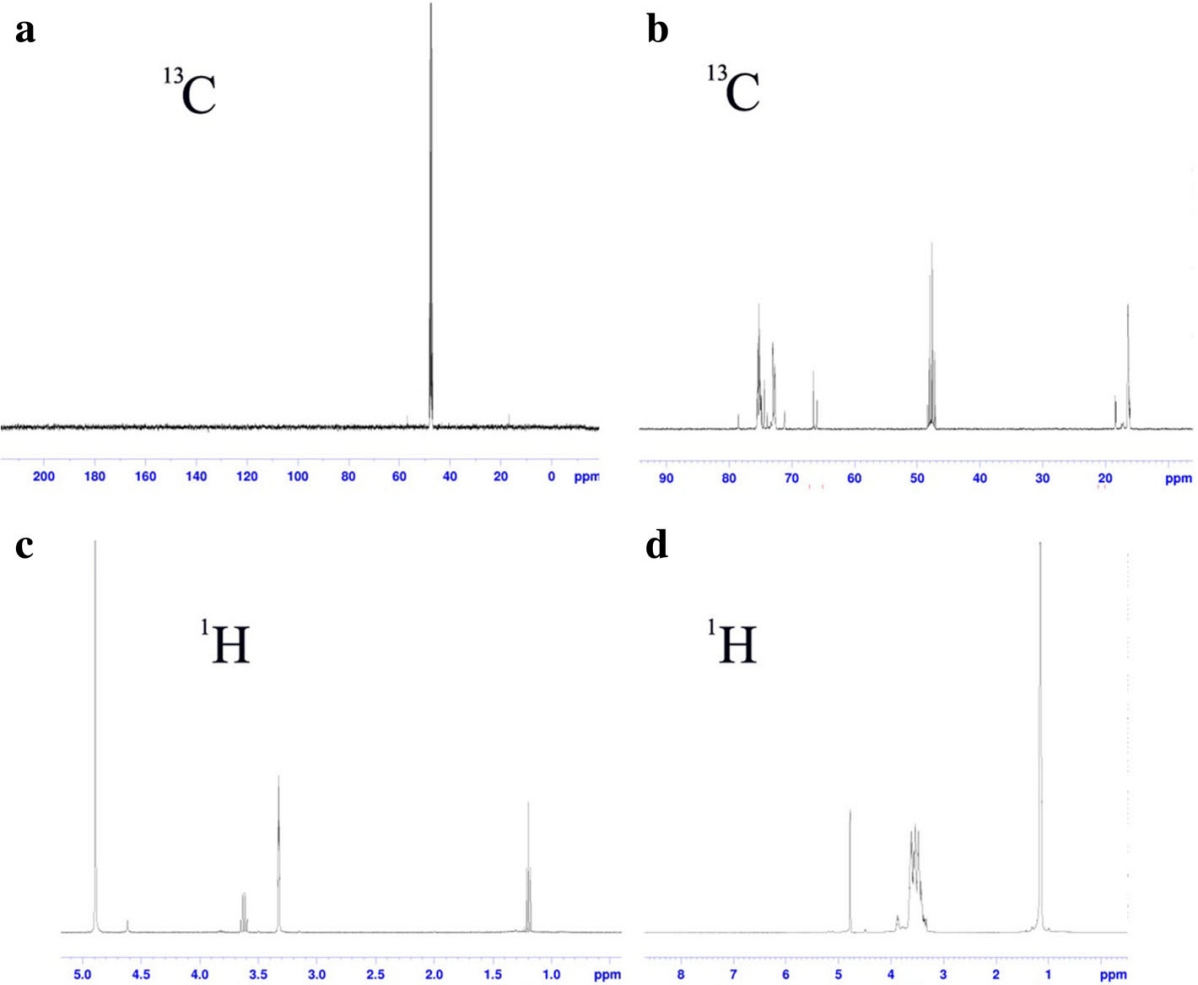
**a** 13C NMR spectra of silica sol C. **b** 13C NMR spectra silica sol D. **c** 1H NMR spectra of c silica sol C. **d** 1H NMR spectra of silica sol D

The ^13^C HMR spectra and ^1^H NMR spectra of silica sols are shown in Fig. 3. For ^13^C HMR, the peak at 48 ppm (Fig. 3a, b) was attributed to the presence of Si–OCH_3_ group in the aerogel network. In addition, the peak at 66 ppm shown in Fig. 3b was attributed to the presence of Si–CH_2_– [31]. This may be due to the addition of PO in the silica sol. In Fig. 3d, the peaks between 3.3 and 3.6 ppm are attributed to Si–OCH_2_–, indicating the presence of PO bonded to the backbone of silica [31–33]. Peaks at 1.6 ppm indicate the presence of Si–CH_3_ in the silica matrix [34–36].

### 3.2 SEM and TEM Characterizations of Dual-layer and Tri-layer Films

**Fig. 4:**
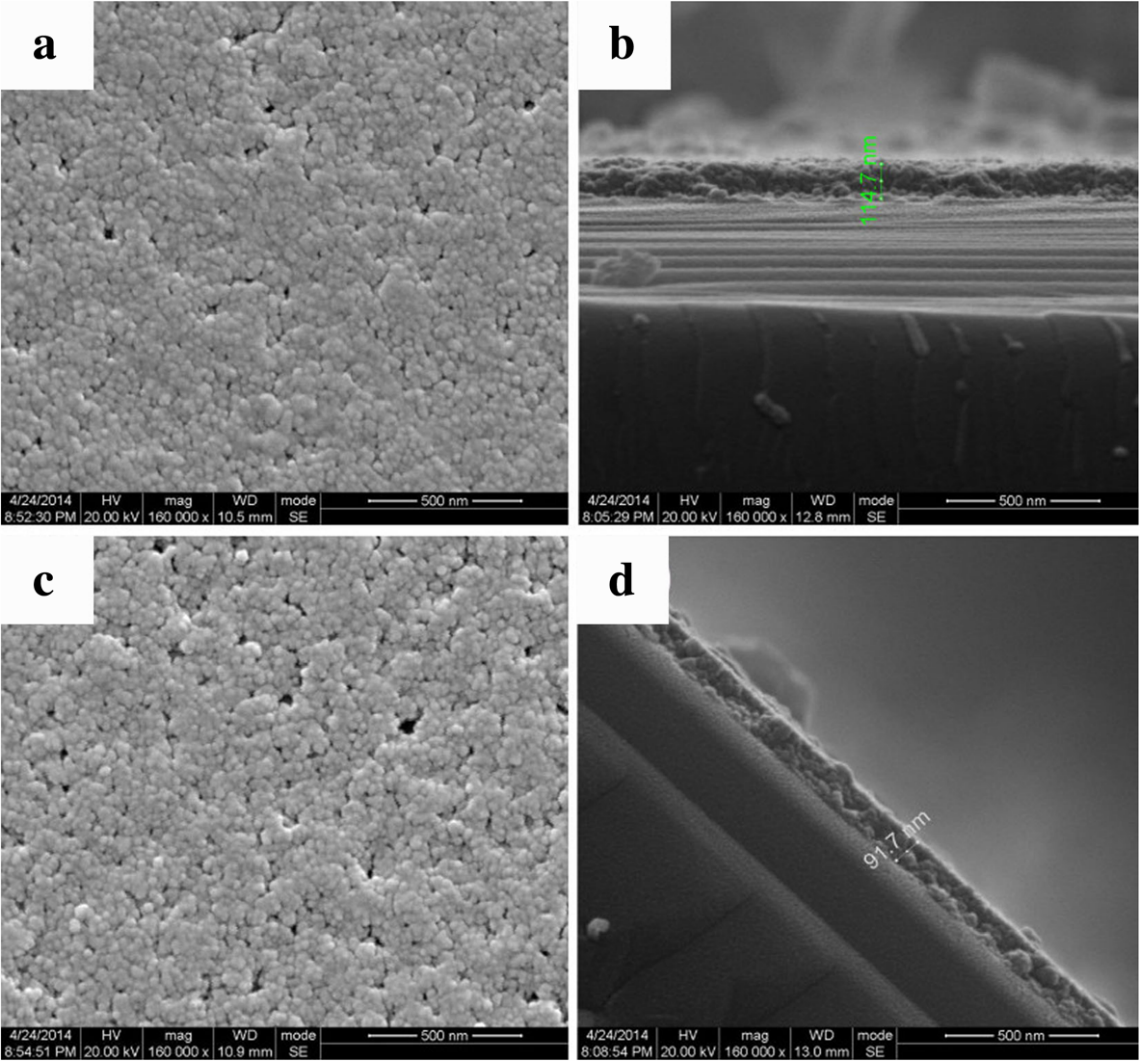
**a** SEM images of sol C based single-layer film. **b** Cross-sectional SEM images of sol C based single-layer film. **c** SEM images of sol D based single layer film. **d** Cross-sectional SEM images of sol D based single layer film

SEM images (Fig. 4a–d) demonstrate the surface morphology and cross-section of single-layer films coated by sol C and sol D, respectively. The thicknesses of both films are uniform. The sol D based film demonstrates a more porous morphology compared to the sol C based film, indicating the PO modification could decrease the density of the film.

**Fig. 5:**
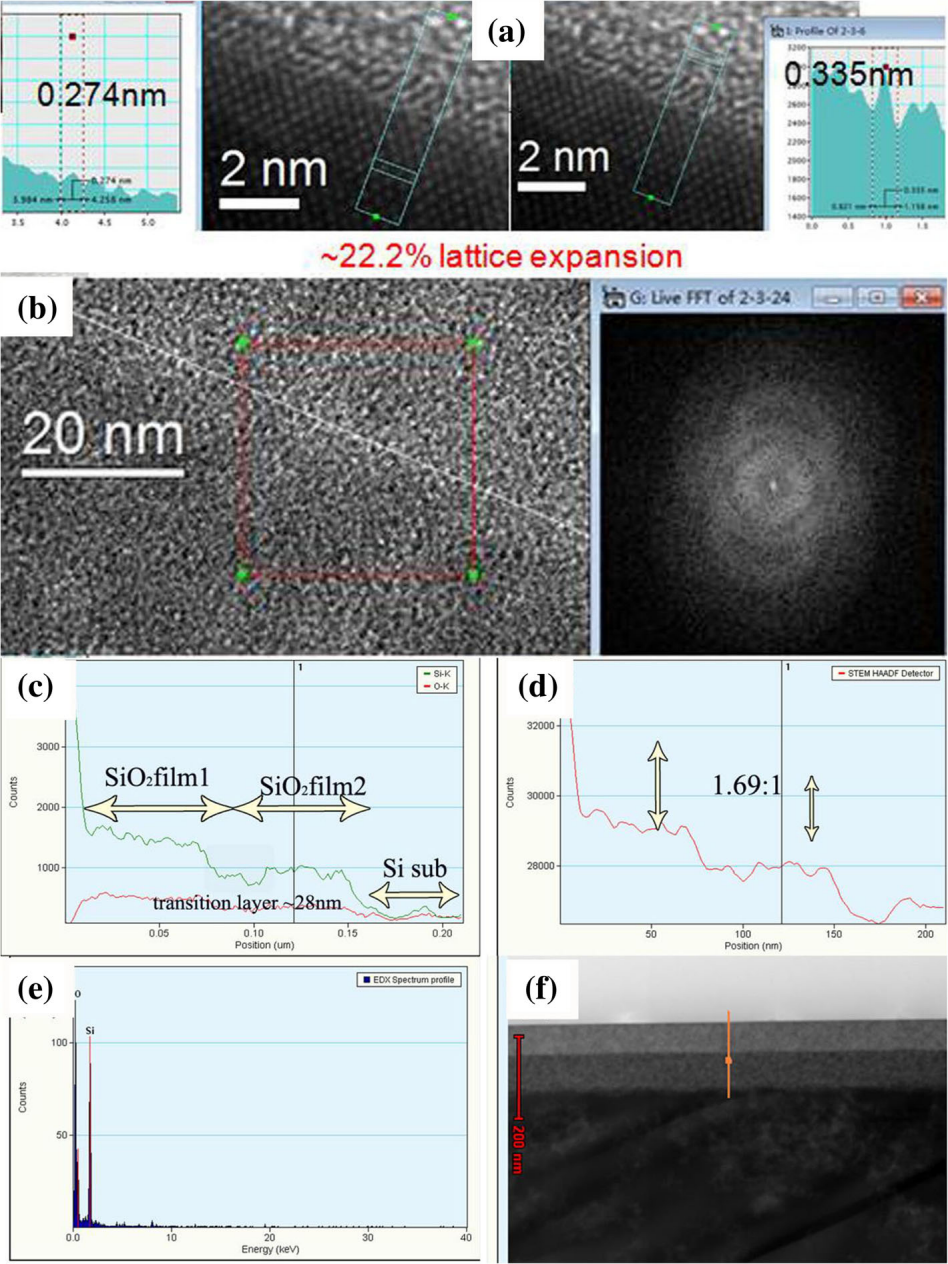
**a** HRTEM images of interfacial area between Si substrate and silica film. **b** TEM images of interfacial area between two layers. Insert is the Fourier transforming spectra. **c**–**e** EDS images of dual-layer silica film via STEM. **f** Cross-sectional TEM images of dual-layer film

TEM is an ideal tool for the investigation of structure in nanoscale, and it can probe more detailed information for the interface. The cross-sectional TEM images of the dual-layer films are shown in Fig. 5. There exists an interfacial area between the bottom layer and the silicon substrate as shown in Fig. 5a. The interface between the top layer and the bottom layer (Fig. 5b) were clear and apparent, which may be due to the mismatch of the two layers due to the difference of density and particle size [35], suggesting that no obvious penetration occurred between the two layers. The inserted FTIR fingerprint spectra indicated the film is amorphous. In Fig. 5f, it can also be seen that the bottom layer was compact, while the top layer was porous (according to the contrast difference). Because the bottom layer was prepared under acidic catalysis conditions and the growth of the silica sol tends to form linear chains and finally grows into strongly cross-linked pore frameworks after calcination. On the other hand, the top layer prepared under basic catalysis conditions had a high volume from the stack of the PO molecules and silica particles. The cross-sectional TEM images and element linear scanning images are shown in Fig. 5c–e, a sharp edge appearing at the position of the interface between two layers. The density ratio is 1.69:1.

**Fig. 6:**
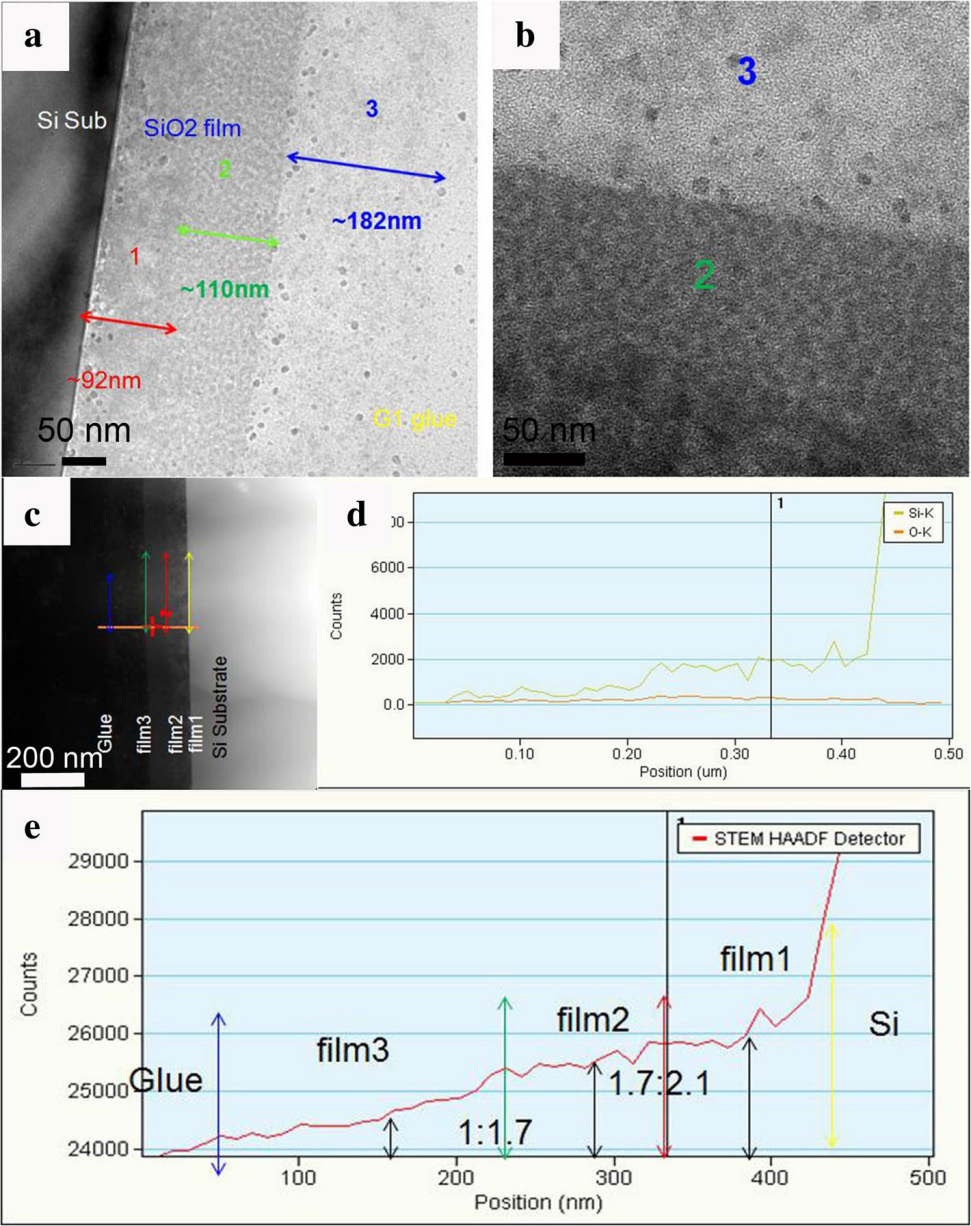
**a** TEM image of the tri-layer films. **b** TEM image of high magnification from the same sample. **c** DF-STEM images of cross-sectional tri-layer film. **d**, **e** EDS images of dual-layer silica film via STEM

The cross-sectional TEM images of the tri-layer film are shown in Fig. 6a, b (high magnification). In Fig. 6a, the sizes of pores in different layers were analyzed. The special pore sizes in bottom, middle, and top layers were 5.1, 7.8, and 10.2 nm, respectively, indicating the density decreased from bottom to top layer. As the density of different layers was distinctively different, the interface can be seen clearly (Fig. 6b). The grain size of silica in the bottom-layer film is smaller and compactly distributed, while the grains in top layer were discretely distributed. The DF-TEM image of the tri-layer film is present in Fig. 6c. The elemental linear scanning images are shown in Fig. 6d–e. The density ratio is 2.1:1.7:1 from bottom to top layer.

### 3.3 Optical Performance of Dual-layer Films

**Fig. 7:**
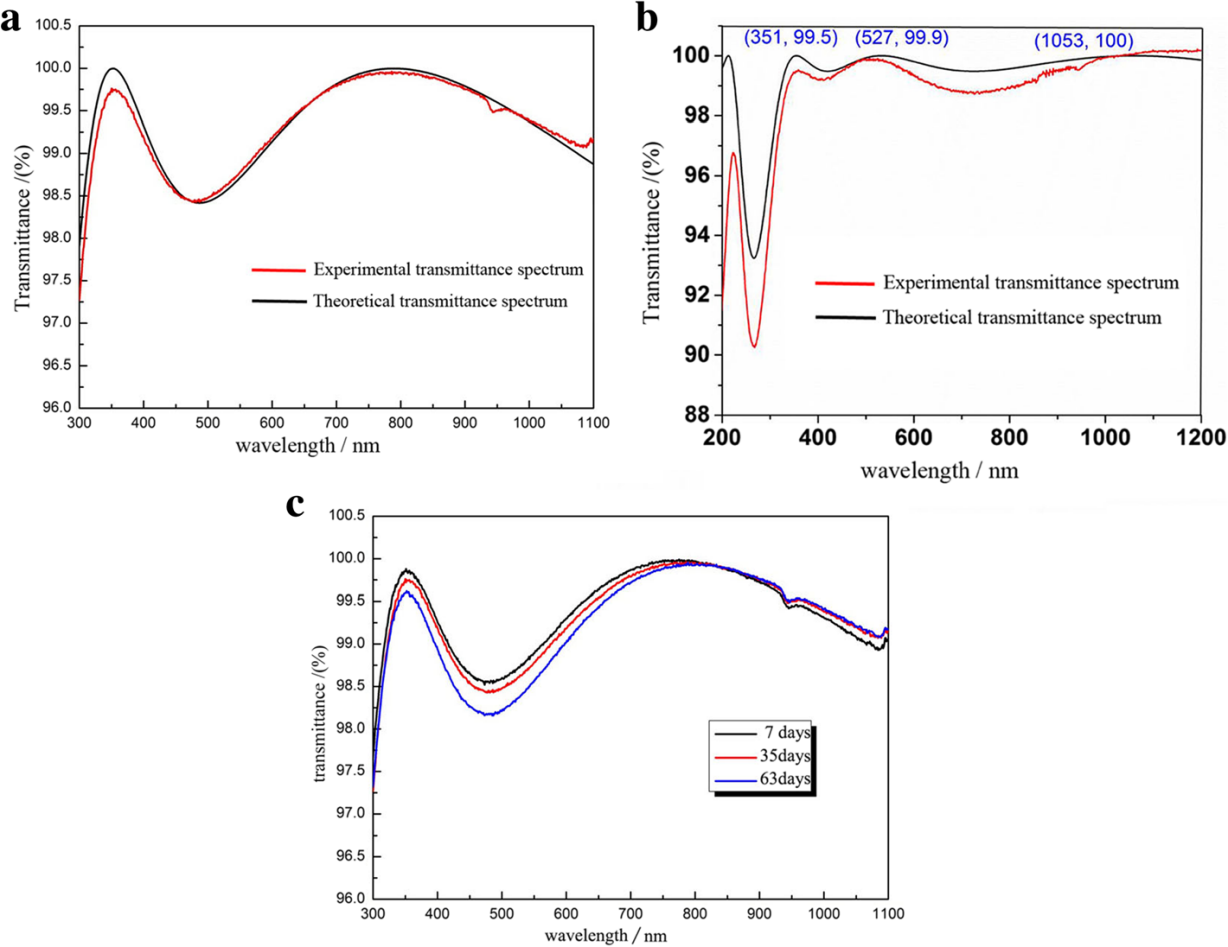
**a** Transmittance spectrum of dual-layer AR film on fused silica substrate. **b** Transmittance spectrum of tri-layer AR film on fused silica substrate. **c** Transmittances of the dual-layer AR films after 7, 35, and 63 days

Figure 7a, b shows the transmittance spectra separately for the dual- and tri-layer AR films on fused silica substrate. The maximum transmittance of the dual-layer AR film was approximated to 99.8% at the central wavelength of 351 nm and nearly 99.5% at the central wavelengths of 1053 nm. For tri-layer AR film, the maximum transmittance reached nearly 100% at both the central wavelengths of 527 and 1053 nm. Moreover, there is no obvious degradation after 63 days for dual-layer AR film (Fig. 7c).

## Conclusions

Dual-/tri-layer broadband AR films were prepared by a sol–gel process. The sols and films were characterized by FTIR, NMR, and TEM. FTIR spectrum indicates that the PO molecules were covalently bonded to the silica particles. The bridge structure existing in PO modified sol contributes to larger silica particles in the layer with low density. Both pore size and grain size demonstrate an increasing trend from bottom layer to top layer. An apparent interface can be observed between each two layers. The density ratios between different layers are measured by cross-sectional STEM. For the dual-layer film, the density ratio of bottom layer and top layer is 1.69:1; for the tri-layer film, the density ratio of bottom layer, middle layer, and top layer is 2.1:1.7:1. The dual-layer AR film shows a good transmittance simultaneously in the wavelengths of 351 and 1053 nm, while the maximum transmittance for tri-layer appeared at 527 and 1053 nm, nearly 100%. Besides, there is no distinctive difference on transmittance after 63 days in terms of the dual-layer AR film.
